# Robust Optimization of GMAW Parameters in 6063–T5 Aluminium Welds Using Taguchi Design, ANOVA, and Monte Carlo Simulation

**DOI:** 10.3390/ma19122499

**Published:** 2026-06-10

**Authors:** José L. Meseguer-Valdenebro, Eusebio José Martínez Conesa, Diego Vergara

**Affiliations:** 1 Escuela Técnica Superior de Ingenieros Industriales, Universidad Politécnica de Cartagena, Member of European University of Technology (EUT+), c/Dr. Fleming s/n, 30201 Cartagena, Spain; joseluis.meseguer@upct.es; 2 ETS de Arquitectura y Edificación, Universidad Politécnica de Cartagena, Member of European University of Technology (EUT+), Edificio CIM, Calle Real, 3, 30201 Cartagena, Spain; eusebio.martinez@upct.es; 3 Technology, Instruction and Design in Engineering and Education Research Group (TiDEE.rg), Facultad de Ciencias y Artes, Universidad Católica de Ávila (UCAV), Calle Canteros s/n, 05005 Ávila, Spain

**Keywords:** Taguchi, weld, aluminium, L9, ANOVA, hardness, Montecarlo, simulation

## Abstract

This study investigates the influence of Gas Metal Arc Welding (GMAW) parameters on the elemental composition and hardness of 6063–T5 aluminum alloy welds. Power, welding speed, and bevel spacing were evaluated using Taguchi’s L9 design to quantify their effects on magnesium and silicon contents and on weld hardness. ANOVA revealed that welding power is the only statistically significant factor governing the incorporation of Mg and Si into the weld metal, whereas no welding parameter showed a statistically significant direct effect on hardness within the investigated range. Nevertheless, an inverse relationship was observed between Mg–Si content and weld hardness, indicating that increased precipitation is associated with lower hardness values. Regression models derived for Mg and Si content were further combined with a Monte Carlo simulation comprising 100,000 iterations, enabling a probabilistic assessment of process robustness. The stochastic analysis confirmed the dominant influence of power on elemental composition and identified a robust operational window ensuring high hardness and controlled Mg–Si levels. The optimal conditions predicted by Taguchi (1596 W, 11.5 mm/s, 1.2 mm) were found to lie within the high-probability region obtained through Monte Carlo simulation. The combined use of Taguchi analysis, ANOVA, regression modelling, and Monte Carlo simulation enabled the evaluation of both process performance and robustness under parameter variability.

## 1. Introduction

Aluminum alloys from the 6xxx series are widely used in transportation, structural engineering, shipbuilding, and lightweight industrial applications because of their favorable combination of mechanical strength, corrosion resistance, and low density. Among them, alloy 6063–T5 is extensively employed in tubular and structural components due to its excellent extrudability and weldability. However, welding of precipitation-hardened aluminum alloys remains a challenging process because thermal cycles generated during welding strongly affect elemental composition changes, distribution of alloying elements, and consequently the mechanical performance of the welded joint.

Gas Metal Arc Welding (GMAW) is one of the most commonly used joining techniques for aluminum alloys because it provides high productivity, automation capability, and acceptable weld quality. Nevertheless, the heat input generated during GMAW significantly influences the formation and redistribution of Mg–Si precipitates within the weld metal and heat-affected zone (HAZ). Variations in heat input may alter grain morphology, precipitate density, dendritic structures, and hardness distribution, ultimately affecting the mechanical integrity of the welded joint.

Similar observations have been reported in previous studies dealing with precipitation-hardened aluminum alloys, where welding heat input plays a decisive role in the final microstructure and mechanical behavior of the joint. Yu et al. [1] demonstrated that both welding heat input and post-weld heat treatment significantly influence the hardness evolution of friction stir welded 2024-T3 aluminum alloys. Their results showed that excessive thermal input can alter precipitate stability within the stir zone, directly affecting local hardness values and the overall mechanical response of the welded joint.

In a similar way, Wang et al. [2] investigated TIG-welded AA6082-T6 aluminum alloys and reported that high heat input promotes porosity formation and microstructural coarsening, leading to reductions in mechanical performance. Their study also highlighted the importance of controlling thermal conditions to preserve precipitate distribution and avoid degradation of the heat-affected zone.

Acharya et al. [3] analyzed friction stir welded AA6092/SiCp-T6 composites and observed that variations in heat input strongly affect compositional variations and precipitate evolution. They concluded that optimized thermal conditions improve both hardness and tensile behavior due to the formation of a more homogeneous microstructure.

Likewise, Yanagihara et al. [4] demonstrated that gas content and heat input have a direct influence on the welding strength of aluminum alloy joints. Their findings confirmed that inadequate thermal control during welding can generate microstructural heterogeneity and weaken the integrity of the weld. Collectively, these studies confirm that heat input is one of the most critical variables governing precipitate evolution, hardness distribution, and mechanical performance in welded aluminum alloys.

The role of alloying elements is also fundamental in determining the mechanical response of aluminum welds. Magnesium and silicon are particularly important because they promote the formation of Mg_2_Si precipitates responsible for precipitation hardening. However, the redistribution and excessive accumulation of these precipitates during welding may produce heterogeneous microstructures and localized hardness reductions. Therefore, understanding the relationship between welding parameters, precipitate evolution, and hardness is essential for process optimization.

Several researchers have applied the Taguchi method and other optimization approaches to improve the quality and mechanical performance of welded joints in aluminum alloys and other engineering materials. Anand et al. [5] employed the Taguchi technique to optimize TIG welding parameters in stainless steel and mild steel joints, demonstrating that the method is highly effective for identifying the most influential welding variables and improving joint strength with a reduced number of experiments. Recent studies have demonstrated the effectiveness of statistical approaches such as Taguchi design and ANOVA for optimizing welding parameters and improving process performance in aluminum alloy welding [6,7].

Goyal et al. [8] investigated the mechanical and microstructural properties of TIG-welded stainless steel joints and highlighted the importance of optimizing process parameters to obtain refined microstructures and improved hardness values. Their results confirmed that welding conditions directly influence grain morphology and the resulting mechanical performance of the weld seam.

In the field of MIG welding, Patil and Waghmare [9] optimized welding parameters to improve the strength of welded joints and reported that heat input control is essential to achieving sound weld quality and minimizing welding defects. Their study emphasized the strong relationship between process parameters and final mechanical properties.

Izeda et al. [10] focused on robotized welding of aluminum alloys using pulsed transfer mode combined with the Taguchi method. Their work demonstrated that statistical optimization techniques are particularly suitable for automated welding systems, where parameter stability and repeatability are critical for ensuring consistent weld quality.

Sahiti et al. [11] applied the Taguchi methodology to optimize the welding process parameters of pure aluminum joints. Their findings showed that the method successfully identifies optimal combinations of process variables capable of enhancing weld strength and reducing process variability.

Ahmad [12] also demonstrated the effectiveness of the Taguchi approach in pulsed TIG welding optimization, highlighting that systematic experimental design can significantly improve weld quality while reducing the number of required experimental trials and associated costs.

In friction stir welding research, Piccini and Svoboda [13] analyzed the influence of tool rotational speed and penetration depth on dissimilar aluminum alloy spot welds. Their study revealed that process parameters strongly affect material flow, heat generation, and ultimately the mechanical integrity of the welded joint.

Similarly, Hsiao et al. [14] combined the Taguchi method with grey relational analysis for the optimization of plasma arc welding parameters. Their work demonstrated that multi-response optimization techniques are effective tools for simultaneously improving several quality indicators, including bead geometry, mechanical performance, and process stability. Collectively, these studies confirm that statistical and optimization methodologies provide reliable frameworks for improving welding quality, identifying critical process variables, and enhancing the robustness of welding operations.

Moreover, most available studies employ deterministic optimization approaches that identify optimal parameter combinations but do not evaluate the robustness of the process against parameter variability and experimental uncertainty. In practical industrial applications, welding parameters are naturally subjected to fluctuations, making probabilistic assessment essential for reliable process optimization.

The optimization of welding parameters in heat-treatable 6xxx aluminum alloys has received considerable attention owing to the sensitivity of these alloys to thermal input and compositional variations during welding [15,16,17]. Previous studies on GMAW welding of 6063–T5 aluminum alloys have mainly focused on weld geometry, thermal cycles, and mechanical performance. However, limited attention has been paid to the simultaneous evaluation of weld metal composition and hardness response under process variability. Therefore, the present work investigates the influence of welding parameters on magnesium and silicon content in the weld metal, together with weld hardness, while integrating deterministic and probabilistic approaches through Taguchi analysis, ANOVA, regression modelling, and Monte Carlo simulation.

The proposed methodology enables the identification of optimum welding conditions and provides an assessment of process robustness under parameter variability. By combining statistical optimization with probabilistic analysis, the approach offers additional insight into the expected variability of magnesium and silicon content and hardness response within the investigated process window. Consequently, the methodology may contribute to a more informed selection of welding parameters for 6063–T5 aluminum alloy joints.

The working hypothesis of this study is that welding power is the dominant process parameter governing the magnesium and silicon content of the weld metal and the resulting hardness response during GMAW of 6063–T5 aluminum alloy. Furthermore, it is hypothesized that the combination of Taguchi analysis, ANOVA, regression modelling, and Monte Carlo simulation can identify robust operating conditions under process variability [18,19].

The proposed methodology provides a more reliable framework for welding optimization by not only identifying optimal parameters but also evaluating their stability and robustness under process variability.

Although previous studies have investigated the optimization of GMAW welding parameters for AA 6063–T5 aluminum alloy using deterministic approaches such as response surface methodology [20], the present work extends this research by integrating Taguchi design, ANOVA, regression modelling, and Monte Carlo simulation to evaluate both process optimization and robustness. In addition, magnesium content, silicon content, and weld hardness are analysed simultaneously within a unified deterministic–probabilistic framework. While previous studies have primarily focused on weld geometry and mechanical performance, the present work combines Taguchi design, ANOVA, regression modelling, and Monte Carlo simulation to simultaneously evaluate magnesium content, silicon content, and weld hardness. This integrated framework enables not only the identification of optimal welding conditions but also the assessment of process robustness under parameter variability.

## 2. Experimental Procedure

### 2.1. Welding Equipment Employed

**Figure 1:**
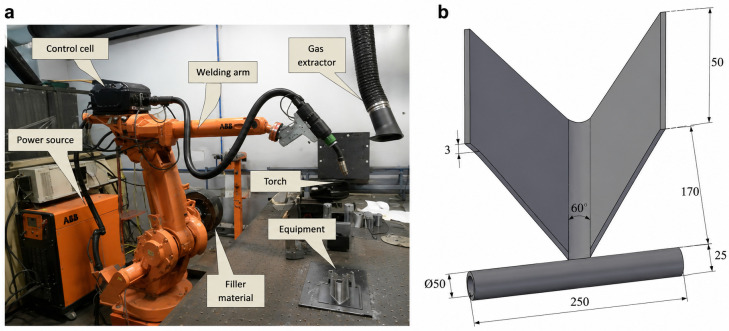
(**a**) Photograph of the welding robot with the GMAW torch installed. (**b**) Tooling used in the welding of the aluminium tube. The welding procedure and specimen preparation were based on the methodology previously reported by Meseguer-Valdenebro et al. [23], with additional experimental and statistical analyses performed in the present study.

The experimental procedure was carried out using a robotic welding arm (ABB, Zurich, Switzerland, model IRB 1400), as shown in Figure 1. The robotic arm has six degrees of freedom and is equipped with a system that controls the welding parameters. The welding robot offers high precision, ensuring proper joint finishing. The elements to be joined are nine aluminum tubes, each with an outer diameter of 50 mm, a wall thickness of 2 mm, and a length of 150 mm. These tubes were cut longitudinally and subsequently joined with a welding bead. To enable the longitudinal joining of the tubes, a carbon steel fixture was designed to hold the two halves of each aluminum tube in place prior to welding. The tooling is shown in Figure 1b.

Aluminum 6063–T5 is typically supplied in tubular form rather than as sheets. The filler metal used is ER5356, which is well-suited for welding 6063–T5 aluminum [21]. The wire used has a diameter of 1 mm, and the shielding gas is 99% pure argon. The welding process employs short-circuit (short arc) metal transfer [22].

### 2.2. Suitability of Specimens Once Welded

**Figure 2:**
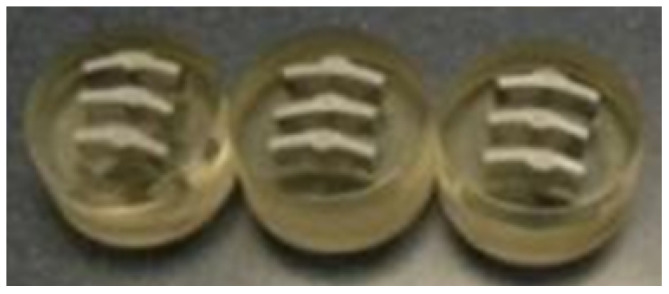
Specimens encapsulated in cold-applied resin.

Once the welding process was completed, the specimens were encapsulated in a cold-curing resin for subsequent metallographic preparation. They were then polished using sandpapers of varying grit sizes to obtain a suitable surface for analysis. The arrangement of the encapsulated specimens is shown in Figure 2.

The welding procedure and specimen preparation were based on the methodology previously reported by Meseguer-Valdenebro et al. [23], with additional experimental and statistical analyses performed in the present study.

### 2.3. Chemical Composition

**Figure 3:**
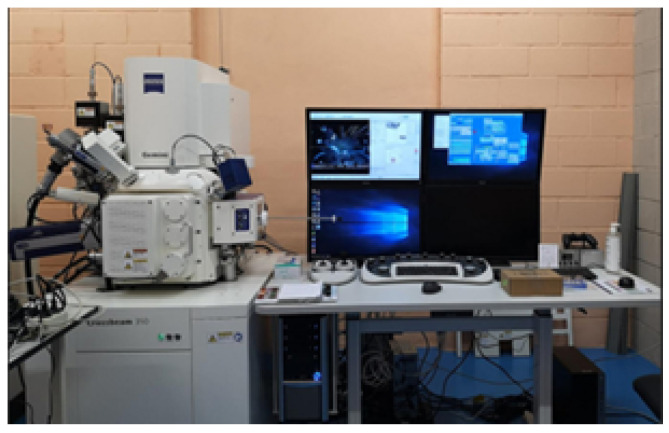
X-ray equipment to measure the chemical composition of the welds analyzed.

The chemical composition of the weld metal was determined using an Xplore 30 X-ray fluorescence (XRF) analyzer (Oxford Instruments plc, Abingdon, UK), as shown in Figure 3. This equipment enables the identification and quantification of the principal alloying elements present in the weld metal, allowing a comparative evaluation of the elemental composition under the different welding conditions investigated.

### 2.4. Chemical Composition of Base Material vs. Filler Material

The chemical compositions of the base metal (alloy 6063–T5) and the filler material (ER5356) are presented in Table 1 and Table 2, respectively. The analysis reveals that the primary difference between the two materials lies in the magnesium content, which is significantly higher in the filler material. This difference, approximately 4.05%, suggests a potential improvement in the mechanical properties of the weld bead, as magnesium acts as a grain refiner, enhancing both strength and ductility.

The remaining elements show minimal or no differences, indicating good chemical compatibility between the base material and the filler material. This similarity helps prevent the formation of unwanted intermetallic phases during the welding process.

As shown in Table 1 and Table 2, magnesium and silicon are the principal alloying elements associated with the objectives of this study. Chromium was detected in the base material at a low concentration but was below the detection limit in the ER5356 filler metal. Owing to its relatively low concentration compared with Mg and Si, chromium is not expected to significantly affect the compositional variations or hardness responses investigated in this work.

**Figure 4:**
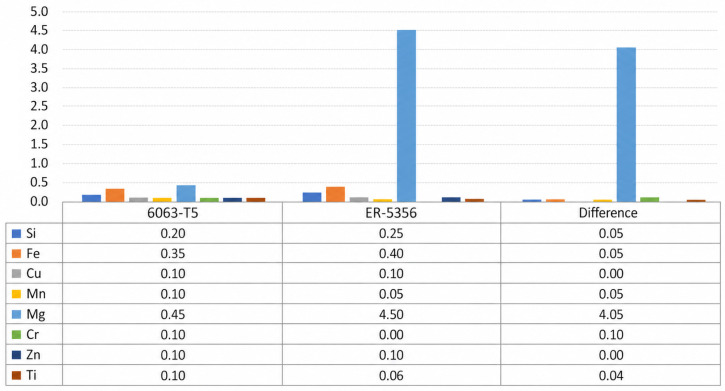
Comparison of the chemical composition between the base metal and the weld metal in the as-supplied condition.

Figure 4 shows that the greatest difference between the filler material and the base material corresponds to the magnesium content, which is 4.05% higher in the filler material. As explained in As previously discussed, magnesium acts as a grain refiner, enhancing the strength and ductility of the material. Therefore, its presence in the weld seam is expected to improve the mechanical properties. The remaining elements do not show significant differences between the base and filler materials.

### 2.5. Experiment Design

Three variables, each at three levels, were used to perform nine welding experiments based on Taguchi’s design, employing an orthogonal L9 matrix. Table 3 presents the selected variables: power (P), welding speed ($v_{s}$), and edge separation (Sp). The levels of each variable are arranged in ascending order. For power, 1557 W represents the minimum level, 1673 W the intermediate level, and 1744 W the maximum level used in the experiments. Similarly, welding speed and edge separation were defined with increasing values corresponding to the first, second, and third levels, respectively.

The statistical software Minitab 21.1.0 was used to construct the matrix of nine experiments following the Taguchi method [24]. Table 4 presents the nine experimental trials proposed using this methodology.

**Figure 5:**
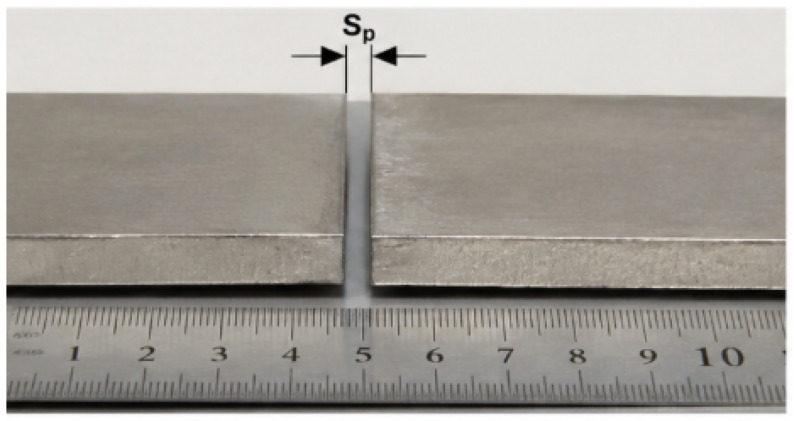
Definition of edge separation (Sp) used in the experimental design.

Figure 5 illustrates the butt-joint configuration employed in the present study. The parameter edge separation (Sp) corresponds to the gap between the facing edges of the two plates before welding. Three levels of edge separation were investigated (0.0, 1.0, and 1.2 mm) in order to evaluate its influence on the chemical composition and hardness of the weld metal.

The power values were calculated as the product of current and voltage. Table 3 shows the current and voltage values corresponding to the three power levels used in the experimental design.

The levels of welding power and welding speed were selected based on preliminary welding trials and previous experience with GMAW welding of 6063–T5 aluminum alloy. The selected ranges correspond to a stable operating window capable of producing defect-free welds while avoiding excessive heat input or lack of fusion. Although the differences between adjacent levels appear moderate, they produce measurable variations in thermal input and consequently in the chemical composition and mechanical properties of the weld metal Table 5.

### 2.6. Hardness Measurements

Hardness measurements were performed on the weld bead using a Vickers hardness tester (Mitutoyo, HM, Kawasaki, Japan). Three indentations were made on each welded specimen within the central region of the weld bead, and the reported hardness value corresponds to the arithmetic mean of the measurements. The indentation locations were selected to provide representative hardness values while minimizing the influence of local variations within the weld metal.

The hardness measurements were used as one of the response variables in the Taguchi optimization study to evaluate the influence of welding power, welding speed, and edge separation on the mechanical performance of the welded joints.

## 3. Results

### EDX Analysis

**Figure 6:**
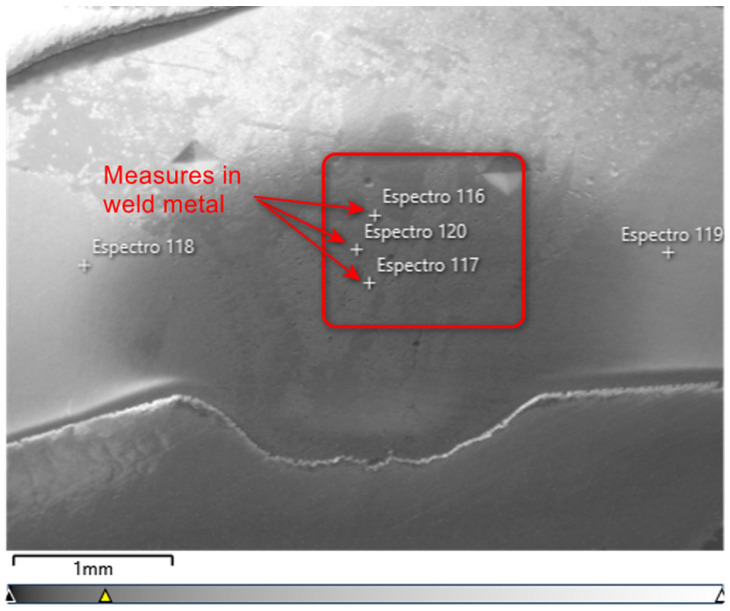
Measurements of the chemical composition in the weld seam of specimen 6.

The chemical composition of the weld metal was determined via Energy-Dispersive X-ray Spectroscopy (EDX) using the equipment shown in Figure 3. Three measurements were performed on each specimen at different locations within the weld bead. As an example, Figure 6 presents the EDX measurements obtained for Specimen 6.

For detailed compositional analysis, Specimen 6 was selected as a representative welding condition. Three measurement locations were chosen across the weld bead to evaluate possible local variations in magnesium and silicon content. Although nine experimental conditions were investigated, the detailed EDX characterization was focused on a representative specimen in order to provide a clear assessment of elemental distribution within the weld metal while maintaining a manageable scope for the compositional analysis.

The EDX analysis focused primarily on magnesium and silicon because these elements are the main alloying constituents associated with the mechanical response of 6063–T5 aluminum alloys. Their distribution within the weld metal was therefore evaluated and correlated with the hardness measurements presented in the following sections.

**Figure 7:**
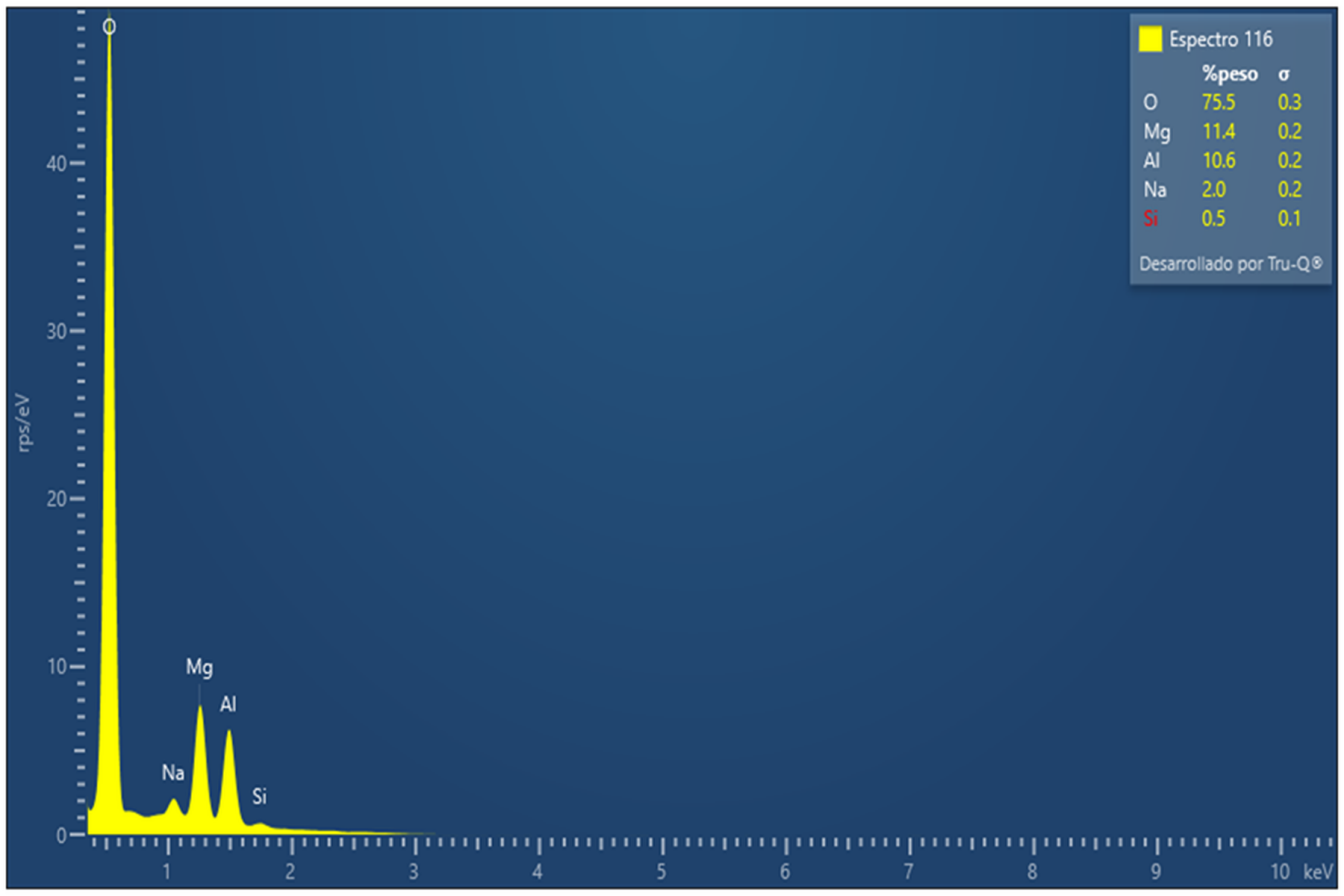
Chemical composition measurements performed on the weld seam of specimen 6.

Figure 7 presents the results of the chemical composition measurements performed on the weld seam of specimen 6, obtained by X-ray diffraction. This figure allows visualization of the distribution of the main alloying elements in the welded area.

The average magnesium and silicon contents measured in the weld metal for the nine experimental conditions are summarized in Table 6.

As shown in Table 6, all magnesium values exceed the nominal magnesium content of the ER5356 filler metal, indicating effective transfer of alloying elements during welding. Furthermore, both magnesium and silicon contents tend to increase with increasing welding power.

As shown in Table 7, the average magnesium content increased from approximately 5.7% at 1557 W to 14.8% at 1744 W, indicating that welding power strongly influences the incorporation of magnesium into the weld metal. A similar trend was observed for silicon, whose average content increased from approximately 0.2% at 1557 W to 1.0% at 1744 W.

To further evaluate the influence of welding power on mechanical performance, hardness measurements were analyzed as a function of power level.

Table 8 shows an increase in the amount of silicon precipitates in the weld bead as the power of the source increases. This behavior suggests that higher power promotes the diffusion and reaction of silicon, contributing to the formation of precipitates that can influence the mechanical and thermal properties of the weld bead.

Table 9 shows an increase in hardness in the weld seam. It is observed that, as the power of the source decreases, the hardness of the weld bead increases.

Taguchi’s method allows for a systematic evaluation of the influence of each welding variable considered in this experiment—power, welding speed, and edge separation—on the responses obtained: the percentage of magnesium, the percentage of silicon, and the hardness of the weld seam. This methodology facilitates the identification of the most significant parameters and their impact on the properties of the resulting material.

## 4. Optimization of the Results by Taguchi’s Method

The Taguchi method was employed to evaluate the influence of welding power (*P*), welding speed ($v_{s}$), and edge separation (Sp) on magnesium content, silicon content, and weld hardness. This methodology assesses process robustness through the signal-to-noise ratio (S/N), which combines the mean response and its variability into a single performance indicator. Depending on the optimization objective, different S/N formulations can be applied. In the present study, the standard Taguchi formulations for the smaller-is-better and larger-is-better criteria were adopted according to the methodology described by Taguchi [25] and Roy [26].

For response variables that must be minimized, the smaller-is-better criterion is expressed as follows:

(1) $$S/N_{i}=-10\cdot \log\left(\frac{1}{n}\sum_{j=1}^{n}Y_{ij}^{2}\right)$$

For response variables that must be maximized, the larger-is-better criterion is defined as follows:

(2) $$S/N_{i}=-10\cdot \log\left(\frac{1}{n}\sum_{j=1}^{n}\frac{1}{Y_{ij}^{2}}\right)$$

where $Y_{ij}$ is the measured response value and *n* is the number of repetitions.

For magnesium and silicon contents, the smaller-is-better criterion was adopted. For hardness, the larger-is-better criterion was selected.

### 4.1. Criteria: Smaller-Is-Better

**Figure 8:**
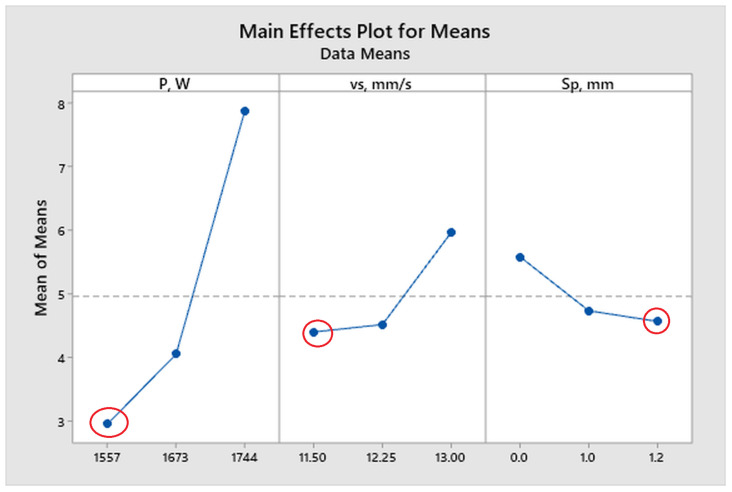
Mean values of smaller silicon and magnesium precipitates.

Figure 8 highlights the lowest mean values of the percentage of magnesium and silicon precipitates (marked with red circles). Based on this analysis, the optimal welding parameters determined for minimizing the formation of these precipitates are as follows:

- P=1557W;
- $V_{s}=11.5\;\mathrm{mm}/s$;
- Sp=1.2mm.

The influence of each welding variable on the formation of silicon and magnesium precipitates is presented in the following table.

In the response tables, Delta represents the difference between the maximum and minimum average response values for each factor. A larger Delta value indicates a greater influence of the corresponding factor on the response variable. The term Rank indicates the relative importance of each factor, with Rank 1 corresponding to the most influential parameter.

The specific weight of each welding variable is presented in Table 10:

- P=66%
- $V_{s}=21\%$
- Sp=14%

Therefore, power is the welding variable that has the greatest influence on magnesium and silicon contents in the weld metal.

### 4.2. Model Fit for Magnesium Content

The analysis of variance (ANOVA) results are shown in Table 11.

In the ANOVA tables, DF denotes the degrees of freedom, Adj SS the adjusted sum of squares, Adj MS the adjusted mean square, F-value the ratio between the factor variance and the residual variance, and *p*-value the probability associated with the statistical significance test. Factors with *p*-values below 0.05 were considered statistically significant at the 95% confidence level.

Table 11 shows that power has a *p*-value of 0.011, making it the most significant variable in the model. In contrast, welding speed ($v_{s}$) and edge separation (Sp) are less statistically significant.

A first-degree polynomial regression model was adopted to describe the relationship between the welding parameters and the response variables. This model was selected because the Taguchi L9 experimental design provides a limited number of experimental observations, making a linear approximation appropriate for identifying the principal effects of the process variables while avoiding overparameterization. The regression equation is as follows:

(3) $$\%\mathrm{Mg}=-89.6+0.0455\;P+2.02\;v_{s}-1.57\;Sp$$

with a coefficient of determination of $R^{2}=85\%$.

**Figure 9:**
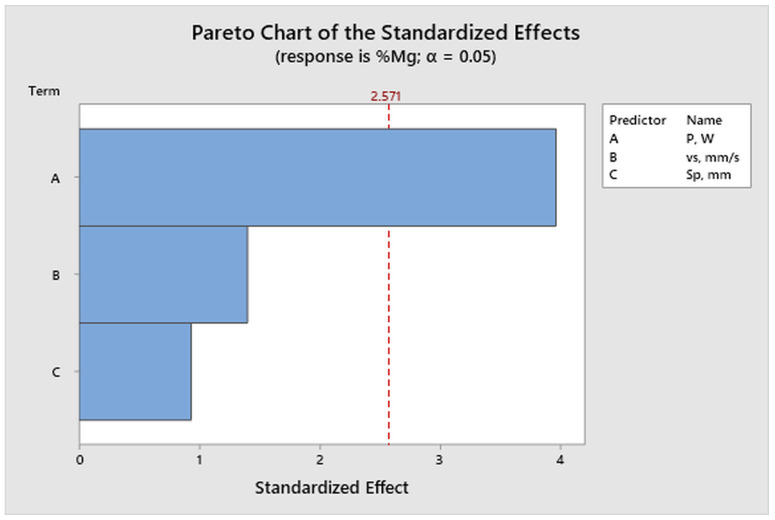
Pareto Chart for Magnesium Percentage.

According to the Pareto chart presented in Figure 9, the power variable exceeds the statistical significance threshold of 0.05, making it the most influential factor in the model. The other variables do not reach this threshold, indicating lower statistical relevance under the evaluated conditions.

### 4.3. Model Fit for Silicon Content

The ANOVA results are presented in Table 12.

According to the results shown in Table 12, the power variable has a *p*-value of 0.008, indicating a statistically significant influence on the percentage of silicon precipitates. In contrast, the welding speed ($v_{s}$) and edge separation (Sp) variables do not reach the established significance level of 0.05, suggesting a lesser impact on the model.

The regression equation obtained is as follows:

(4) $$\%\mathrm{Si}=-6.83+0.00397\;P+0.067\;v_{s}-0.126\;Sp$$

with a coefficient of determination of $R^{2}=88\%$.

**Figure 10:**
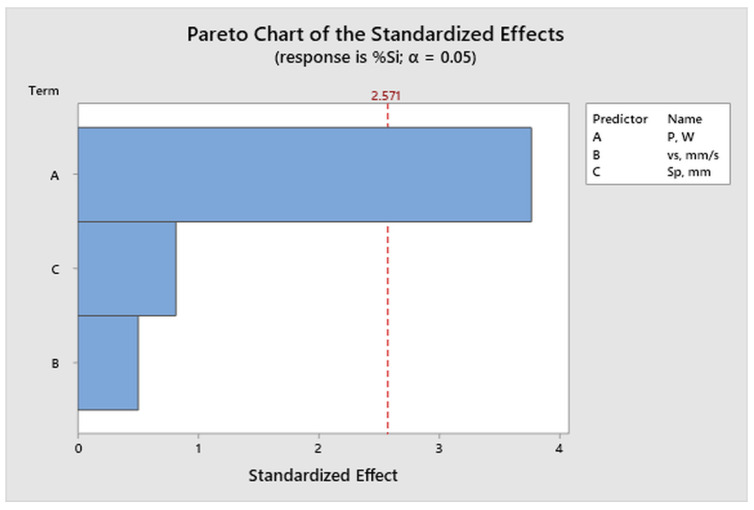
Pareto Chart for Silicon Percentage.

According to the Pareto diagram shown in Figure 10, power is the most significant variable, exceeding the significance threshold of 0.05.

Criteria: the bigger is the better

**Figure 11:**
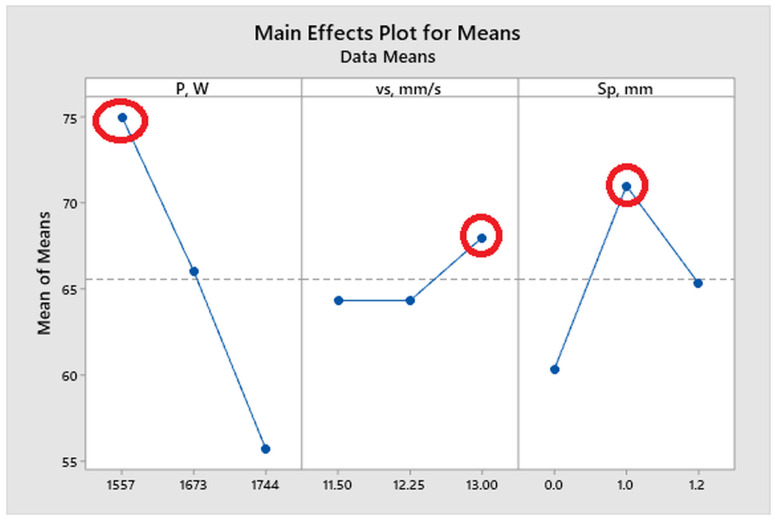
Average values of higher hardness.

Figure 11 highlights the highest average hardness values with red circles. Based on these results, the optimal welding parameters are determined as follows:

- P=1557W;
- $V_{s}=13\;\mathrm{mm}/s$;
- Sp=1mm.

The influence of each welding variable on hardness values is presented in the Table 13.

The results presented in Table 13 indicate that welding power has the strongest influence on hardness, accounting for approximately 57% of the total variation. As welding power increases, the average hardness decreases from 75 HV at Level 1 to 55.67 HV at Level 3. This trend suggests that higher heat input may adversely affect the hardness response of the weld metal.

A possible explanation is that increasing welding power increases the thermal exposure of the weld region, promoting a more homogeneous distribution of alloying elements and reducing localized compositional gradients. Although detailed microstructural characterization was beyond the scope of the present work, the results indicate that thermal effects associated with higher heat input play a significant role in the observed hardness reduction.

In contrast, welding speed and edge separation exhibit considerably lower contributions (11% and 32%, respectively), indicating that their influence on hardness is secondary compared with the effect of welding power.

### 4.4. Adjustment of the Model for the Hardness Values Obtained

The analysis of variance (ANOVA) results are as follows:

Table 14 shows that none of the variables are significant in the model, as all have *p*-values greater than 0.05.

Linear regression analysis was not performed because none of the variables are significant; therefore, the model is not representative.

### 4.5. Correlation Between Hardness and Magnesium and Silicon Contents

**Figure 12:**
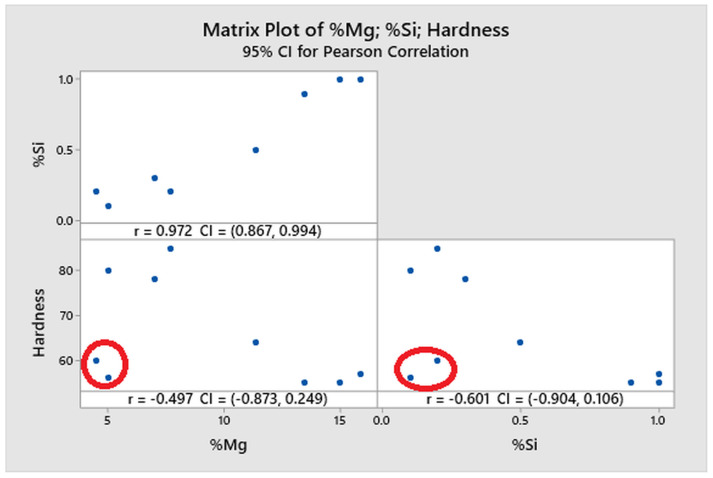
Correlation between Hardness and Si and Mg precipitates.

The correlation between hardness and silicon and magnesium precipitates was determined. Figure 12 shows a clear correlation between silicon and magnesium precipitates. By excluding the two values marked in red, a relationship between magnesium and silicon contents and hardness is identified, indicating that as hardness increases, the number of precipitates decreases.

**Figure 13:**
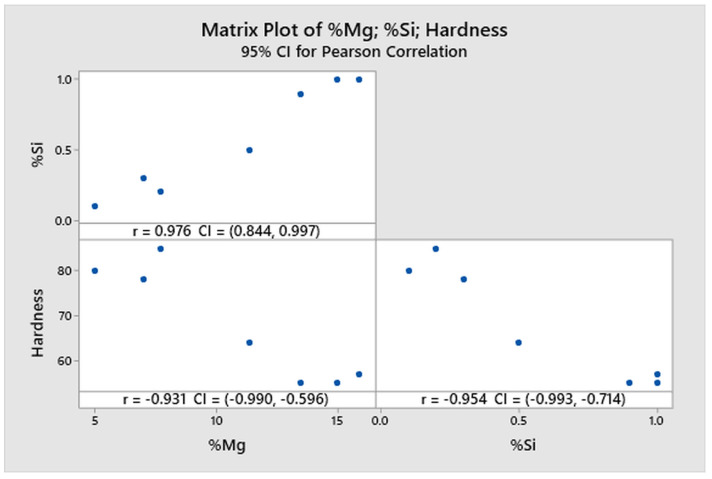
Correlation between Hardness and Si and Mg precipitates with eliminated values.

By excluding these two points, the correlation changes, revealing a clearer relationship between hardness and precipitates (see Figure 13).

### 4.6. Optimized Combination of Welding Parameters

**Figure 14:**
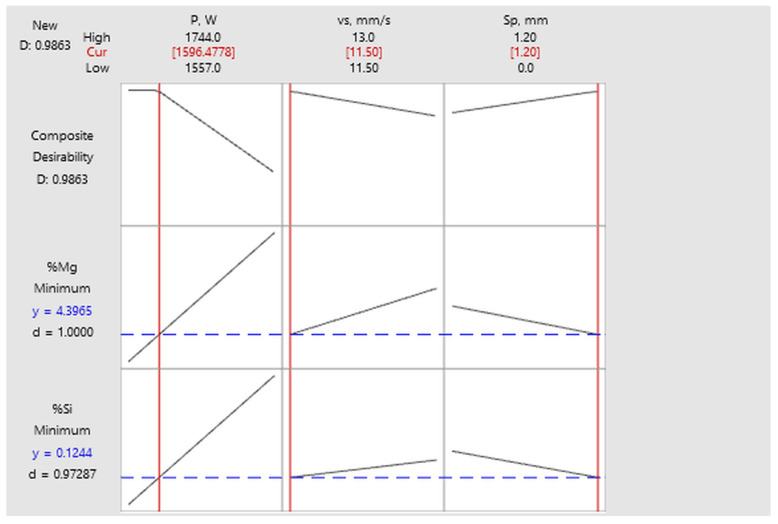
Optimised combination of process parameter.

Once the influence of each input variable on the formation of silicon and magnesium precipitates has been determined, the optimal combination of process parameters is shown in Figure 14.

The optimization graph illustrates the effect of each input variable, represented by columns, on the measurements taken from the weld seam, represented by the responses in rows. Vertical red lines indicate the current settings for each input variable, with their values displayed at the top of each column. Conversely, horizontal blue lines and their corresponding numbers represent the response values obtained at the current level of each factor.

Figure 14 presents the multi-response optimization obtained using the Taguchi methodology. The composite desirability reached a value of 0.98, indicating a parameter combination close to the optimum solution for the selected responses.

The optimal values are as follows:

$$P=1596\;W,\;v_{s}=11.5\;\mathrm{mm}/s,\;Sp=1.2\;\mathrm{mm}.$$

The confidence and prediction intervals obtained from the optimization process are presented in Table 15.

## 5. Monte Carlo-Based Optimization

Although the Taguchi method provides an efficient framework for identifying the most influential welding parameters, it does not explicitly account for the inherent variability of the GMAW process nor for measurement uncertainty in hardness and chemical composition. To complement the deterministic analysis, a Monte Carlo simulation was conducted to evaluate the robustness of the optimal welding conditions.

### 5.1. Methodology

The simulation was based on the regression models obtained for magnesium and silicon content (Equations (3) and (4)). Since hardness did not present statistically significant trends, it was modeled as a normally distributed variable using the experimental mean and standard deviation.

The welding parameters were treated as uniformly distributed variables within the experimental range:

$$P∼U\left(1557,1744\right)\;W,\;v_{s}∼U\left(11.5,13.0\right)\;\mathrm{mm}/s,\;Sp∼U\left(0.0,1.2\right)\;\mathrm{mm}.$$

A total of 100,000 iterations were performed. In each iteration, the responses were computed as follows:

$$\%Mg=-89.6+0.0455P+2.02v_{s}-1.57Sp,$$

$$\%Si=-6.83+0.00397P+0.067v_{s}-0.126Sp,$$

$$H∼N(\mu_{H},\sigma_{H}),$$

where $\mu_{H}=65.6\;\mathrm{HV}5$ and $\sigma_{H}=11.1\;\mathrm{HV}5$ were obtained from the experimental hardness values.

### 5.2. Monte Carlo Simulation Procedure

The Monte Carlo simulation was implemented using Python 3.14, and the resulting probability distributions were subsequently compared with the optimal conditions identified through the Taguchi analysis.

The Monte Carlo simulation was performed using the regression equations obtained for magnesium and silicon content. The welding parameters, namely power (*P*), welding speed ($v_{s}$), and edge separation (Sp), were treated as input variables and randomly sampled within the experimental ranges defined by the Taguchi design.

For each generated combination of input parameters, the corresponding magnesium and silicon contents were calculated using the regression models. The resulting distributions were subsequently analyzed to evaluate process variability and identify the most probable operating conditions.

The simulation was implemented using Python 3.14 using the NumPy for random sampling and data processing and Matplotlib 3.10 libraries, and the obtained probability distributions were compared with the optimum conditions identified through the Taguchi analysis.

### 5.3. Simulation Results

Monte Carlo predictions confirmed the strong dependence of both magnesium and silicon precipitation on welding power. The output distributions showed that:

- For P>1650 W, both %Mg and %Si exhibited a strong upward shift, reproducing the experimentally observed tendency toward increased precipitation.
- Hardness values above 70 HV5 appeared predominantly when P<1620 W, $v_{s}>12.5$ mm/s, and Sp≈1.0−1.2 mm.

A stable operating region was defined as the set of input parameters yielding hardness values above 70 HV5 in at least 75% of simulations and maintaining %Mg ≤8% and %Si ≤0.3%. This region corresponds to the following:

$$1560\leq P\leq 1605\;W,\;12.5\leq v_{s}\leq 13.0\;\mathrm{mm}/s,\;1.0\leq Sp\leq 1.2\;\mathrm{mm}.$$

These results closely match the optimum predicted by the Taguchi method (P=1596 W, $v_{s}=11.5$ mm/s, Sp=1.2 mm), confirming that the selected values lie within the region of highest probability of achieving favorable mechanical performance.

### 5.4. Discussion

Monte Carlo simulation provides a probabilistic insight into the welding process that complements the deterministic Taguchi design. This method allows identification not only of an optimal point but also of a robust process window. The analysis confirms that power is the dominant parameter controlling precipitation behavior, while welding speed and edge separation have secondary but stabilizing roles. The probabilistic nature of the simulation enhances confidence in the selected parameter combination and validates the experimental observations.

### 5.5. Monte Carlo Output Distributions

**Figure 15:**
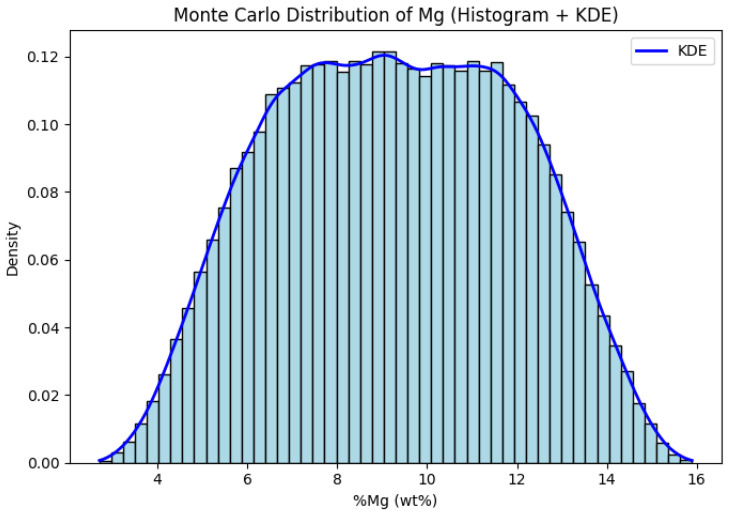
Monte Carlo distribution of magnesium content in the weld bead.

**Figure 16:**
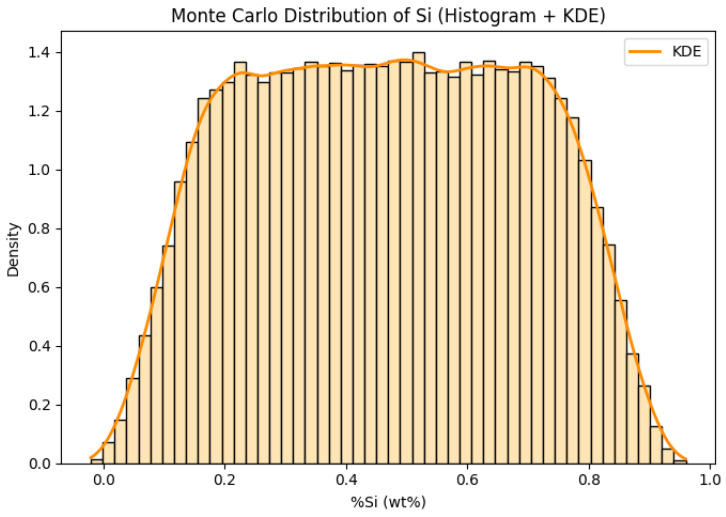
Monte Carlo distribution of silicon content in the weld bead.

**Figure 17:**
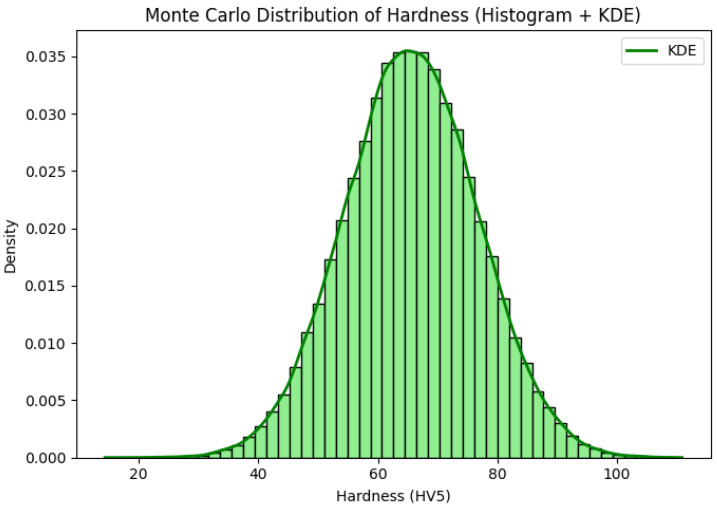
Monte Carlo distribution of hardness values (HV5).

To visualize the probabilistic behavior of the magnesium content, silicon content, and hardness predicted by the Monte Carlo simulation, histograms were generated from 100,000 simulated iterations. These histograms illustrate the expected distribution of each response and provide insight into the robustness of the optimal welding parameters. Figure 15, Figure 16 and Figure 17 show the distributions obtained for %Mg, %Si, and hardness, respectively.

Figure 15 shows the probability distribution of magnesium content obtained from the Monte Carlo simulation. The distribution exhibits a broad central plateau between approximately 7 and 12 wt%, with a progressive reduction in probability towards the lower and upper limits. This behaviour indicates that most combinations of welding parameters lead to intermediate magnesium contents, whereas extreme values occur with lower probability. The distribution is consistent with the dominant influence of welding power identified by the ANOVA analysis and confirms that magnesium incorporation remains within a relatively stable range throughout the investigated parameter space.

Figure 16 presents the probability distribution of silicon content. A similar trend to that observed for magnesium can be identified, with most simulated values concentrated in the intermediate region of the distribution and a lower frequency of occurrence near the boundaries. This result suggests that silicon content is less sensitive to random variations in the input parameters within the investigated operating window. The relatively smooth distribution also supports the robustness of the regression model used in the Monte Carlo analysis.

Figure 17 illustrates the probability distribution of hardness values obtained from the Monte Carlo simulation. Unlike the elemental distributions, hardness exhibits an approximately bell-shaped distribution centred around 65–70 HV5. The concentration of values around the mean indicates that the majority of parameter combinations produce comparable hardness levels, while very low and very high hardness values occur with relatively low probability. This behaviour demonstrates that weld hardness is less sensitive to random parameter fluctuations than the elemental composition and supports the identification of a robust operating region capable of maintaining satisfactory mechanical performance.

Overall, the Monte Carlo simulation confirms that the optimal welding conditions identified by the Taguchi analysis are located within a statistically robust region of the design space. The distributions obtained for magnesium content, silicon content, and hardness indicate that the process remains relatively stable under parameter variability, with most simulated outcomes concentrated around the desired response values. These results reinforce the suitability of the proposed operating window for industrial applications.

### 5.6. Limitations of the Study

The present study was based on Energy-Dispersive X-ray Spectroscopy (EDX) measurements and hardness testing. Although variations in magnesium and silicon content were identified and correlated with the mechanical response of the welds, no metallographic, SEM, or TEM analyses were performed. Consequently, the discussion is limited to compositional variations and hardness behavior, and no direct conclusions regarding precipitate morphology, grain refinement, or microstructural evolution can be drawn. Future work should incorporate detailed microstructural characterization to further investigate the mechanisms responsible for the observed trends.

## 6. Conclusions

This study investigated the influence of welding power, welding speed, and edge separation on the magnesium content, silicon content, and hardness of GMAW welds produced in 6063–T5 aluminum alloy. The analysis combined Taguchi optimization, ANOVA, regression modelling, and Monte Carlo simulation to evaluate both process performance and robustness. The main conclusions are summarized as follows:

1. This study demonstrates the applicability of an integrated deterministic–probabilistic framework for welding process optimization, combining experimental design, statistical modelling, and uncertainty analysis.
2. Welding power was identified as the dominant process parameter affecting both magnesium and silicon content in the weld metal. ANOVA demonstrated that power was the only statistically significant factor, whereas welding speed and edge separation exhibited comparatively minor effects within the investigated range.
3. The developed regression models provided a satisfactory description of the relationship between welding parameters and elemental composition, with coefficients of determination exceeding 85%.
4. Hardness analysis revealed that welding power has the greatest influence on weld hardness. Higher power levels were associated with lower hardness values, suggesting that thermal input plays an important role in the hardness response of the welded joints.
5. Monte Carlo simulation confirmed the deterministic results obtained through the Taguchi methodology and identified a high-probability operating region associated with stable magnesium and silicon contents and favorable hardness values.
6. The optimal welding conditions predicted by the Taguchi analysis were located within the high-probability region identified through Monte Carlo simulation, demonstrating consistency between deterministic and probabilistic approaches.
7. The combined use of Taguchi analysis, ANOVA, regression modelling, and Monte Carlo simulation enabled the evaluation of both process performance and robustness under parameter variability.

## Figures and Tables

**Table 1:** Chemical composition of base metal 6063–T5 (wt.%).

Si	Fe	Cu	Mn	Mg	Cr	Zn	Ti
0.2–0.6	0.35	0.10	0.10	0.45–0.90	0.10	0.10	0.10

**Table 2:** Chemical composition of filler metal ER5356 (wt.%).

Si	Fe	Cu	Mn	Mg	Cr	Zn	Ti
0.25	0.40	0.10	0.05	4.5	–	0.10	0.06

**Table 3:** Factor levels used in the Taguchi L9 design and corresponding welding parameters [23].

Level	Power (W)	Current (A)	Voltage (V)	$v_{s}$ (mm/s)	Sp (mm)
1	1557	90	17.3	11.5	0.0
2	1673	94	17.8	12.25	1.0
3	1744	98	17.8	13.0	1.2

**Table 4:** Matrix of nine experiments based on the Taguchi L9 orthogonal array [23].

Experiment No.	Power (W)	$v_{s}$ (mm/s)	Sp (mm)
1	1557	11.5	0.0
2	1557	12.25	1.0
3	1557	13.0	1.2
4	1673	11.5	1.0
5	1673	12.25	1.2
6	1673	13.0	0.0
7	1744	11.5	1.2
8	1744	12.25	0.0
9	1744	13.0	1.0

**Table 5:** Intensity and voltage values corresponding to each power level [23].

Power (W)	Intensity (A)	Voltage (V)
1557	90	17.3
1673	94	17.8
1744	98	17.8

**Table 6:** Magnesium % weight measurements.

	Test Tube								
% Mg	**1**	**2**	**3**	**4**	**5**	**6**	**7**	**8**	**9**
	4.5	5	7.7	7	5	11.4	13.5	15.9	15
% Si	**1**	**2**	**3**	**4**	**5**	**6**	**7**	**8**	**9**
	0.2	0.1	0.2	0.3	0.1	0.5	0.9	1	1

**Table 7:** Relationship between power level and magnesium content in the weld metal.

Sample	Power (W)	% wt Mg	Average %Mg
1	1557	4.5	5.7
2		5.0	
3		7.7	
4	1673	7.0	7.8
5		5.0	
6		11.4	
7	1744	13.5	14.8
8		15.9	
9		15.0	

**Table 8:** Relationship between power level and silicon content in the weld metal.

Sample	Power (W)	% wt Si	Average %Si
1	1557	0.2	0.2
2		0.1	
3		0.2	
4	1673	0.3	0.3
5		0.1	
6		0.5	
7	1744	0.9	1.0
8		1.0	
9		1.0	

**Table 9:** Relationship between hardness and power levels.

Sample No.	Hardness (HV5)	Average Hardness (HV5)	Power (W)
1	60	75	1557
2	80		
3	85		
4	78	66	1673
5	56		
6	64		
7	55	56	1744
8	57		
9	55		

**Table 10:** Response table for means for Si and Mg.

Level	P (W)	$v_{s}$ (mm/s)	Sp (mm)
1	2.950	4.400	5.583
2	4.050	4.517	4.733
3	7.883	5.967	4.567
Delta	4.933	1.567	1.017
Rank	1	2	3

**Table 11:** Analysis of variance (ANOVA) for magnesium content.

Source	DF	Adj SS	Adj MS	F-Value	*p*-Value
Regression	3	130.556	43.519	6.18	0.039
P (W)	1	110.642	110.642	15.70	0.011
$v_{s}$ (mm/s)	1	13.802	13.802	1.96	0.221
Sp (mm)	1	6.112	6.112	0.87	0.394
Error	5	35.226	7.045		
Total	8	165.782			

**Table 12:** Analysis of variance (ANOVA) for Silicon Content.

Source	DF	Adj SS	Adj MS	F-Value	*p*-Value
P (W)	2	1.10222	0.551111	124.00	0.008
$v_{s}$ (mm/s)	2	0.04222	0.021111	4.75	0.174
Sp (mm)	2	0.04222	0.021111	4.75	0.174
Error	2	0.00889	0.004444		
Total	8	1.19556			

**Table 13:** Response table for means for hardness.

Level	P (W)	$v_{s}$ (mm/s)	Sp (mm)
1	75.00	64.33	60.33
2	66.00	64.33	71.00
3	55.67	68.00	65.33
Delta	19.33	3.67	10.67
Rank	1	3	2

**Table 14:** Analysis of variance (ANOVA) for hardness.

Source	DF	Adj SS	Adj MS	F-Value	*p*-Value
P (W)	2	561.56	280.78	1.39	0.418
$v_{s}$ (mm/s)	2	26.89	13.44	0.07	0.937
Sp (mm)	2	170.89	85.44	0.42	0.702
Error	2	402.89	201.44		
Total	8	1162.22			

**Table 15:** Confidence and prediction intervals obtained from the optimization process.

Response	Fit	SE Fit	95% CI	95% PI
%Mg	2.60	1.98	(−2.49; 7.69)	(−5.91; 11.11)
%Si	−0.032	0.182	(−0.500; 0.435)	(−0.814; 0.749)

PI: Prediction Interval; CI: Confidence Interval.

## Data Availability

The original contributions presented in this study are included in the article. Further inquiries can be directed to the corresponding author.

## Abbreviations

The following abbreviations are used in this manuscript:

Abbreviation	Definition
Adj MS	Adjusted Mean Square
Adj SS	Adjusted Sum of Squares
ANOVA	Analysis of Variance
BM	Base Metal
Delta	Difference between maximum and minimum response means
DF	Degrees of Freedom
DOE	Design of Experiments
F-value	Fisher Test Statistic
ER5356	Aluminum Filler Metal ER5356
FZ	Fusion Zone
GMAW	Gas Metal Arc Welding
GTAW	Gas Tungsten Arc Welding
HAZ	Heat-Affected Zone
HV	Vickers Hardness
MCS	Monte Carlo Simulation
*p*-value	Probability Value
Rank	Relative Importance of Factor
RSM	Response Surface Methodology
SEM	Scanning Electron Microscopy
S/N	Signal-to-Noise Ratio
TEM	Transmission Electron Microscopy
XRF	X-ray Fluorescence
Sp	Edge Separation
*P*	Welding Power
$v_{s}$	Welding Speed
